# MUC15 acts as a tumor suppressor gene which correlates with prognosis and immune infiltration in esophageal squamous cell carcinoma

**DOI:** 10.7150/ijms.108926

**Published:** 2025-03-21

**Authors:** Xiang Fei, Ai-li Wang, Hao Wu, Si-wei Xing, Lei Chen, Ying Chen, Xue-jing Lin, Hai-long Liu, Bin Sun

**Affiliations:** 1 Department of Thoracic Surgery, Shanghai Pulmonary Hospital, School of Medicine, Tongji University, 507 Zhengmin Road, Shanghai 200433, P. R. China.; 2 Center for Clinical Research and Translational Medicine, Yangpu Hospital, Tongji University School of Medicine, 450 Tengyue Road, Shanghai, 200090, P. R. China.; 3 Department of General Surgery, Yangpu Hospital, Tongji University School of Medicine, 450 Tengyue Road, Shanghai, 200090, P. R. China.; 4 Shanghai Municipal Hospital of Traditional Chinese Medicine, Shanghai University of Traditional Chinese Medicine, 274 Zhijiang Middle Road, Shanghai, 200071, P. R. China.; 5 Department of Thoracic Surgery, Changhai Hospital, Naval Medical University, 168 Changhai Road, Shanghai, 200433, P. R. China.; 6 Department of Urology, Ruijin Hospital, Shanghai Jiao Tong University School of Medicine, 197 Ruijin 2nd Road, Shanghai, 200025, P. R. China.

**Keywords:** MUC15, tumor suppressor gene, Weighted co-expression network analysis, prognosis, immune infiltration, esophageal squamous cell carcinoma

## Abstract

Esophageal squamous cell carcinoma (ESCC), as a common malignant tumor of the digestive system, has been a challenge in improving and prolonging the postoperative survival of patients. This study aims to identify novel biomarkers that can guide the clinical diagnosis and treatment by using bioinformatics methods. The RNA-seq data and corresponding clinical data of ESCC were downloaded from the TCGA and GEO database. Weighted co-expression network analysis (WGCNA) was used to identify candidate biomarkers. The LASSO analysis was performed to classify the biomarkers. ROC curve and AUC were used to evaluate the sensitivity and specificity of biomarkers. CIBERSORT was applied to estimate the relative abundances of immune cell types through gene expression profiling. Univariate and multiple Cox regression were performed to screen out prognostic factors. MUC15, which abnormally expressed in different physiological processes and participates in inhibiting or promoting function in different types of tumors, was selected of candidate biomarker. Finally, we validated the expression of MUC15 in ESCC tissues and its inhibitory effect on cell function through *in vitro* and *in vivo* experiments. In conclusion, we identified MUC15 could serve as a tumor suppressor gene and may become a promising candidate for individualized clinical diagnosis and treatment of ESCC.

## 1. Introduction

Esophageal cancer (EC) is a common tumor of the human digestive system, and the incidence rate ranks seventh among malignant tumors. According to the latest statistical data, more than 470000 new cases were diagnosed every year all over the world 1. EC mainly includes two subtypes: esophageal adenocarcinoma (EAC) and esophageal squamous cell carcinoma (ESCC), each with its own unique epidemiological characteristics. ESCC accounts for about 90% of global cases which commonly occurring in South America, East Africa, sub-Saharan Africa, and Central Asia. ESCC is closely related to alcohol intake, tobacco use, dietary habits, and environmental factors in terms of etiology 2-4. A considerable number of ESCC patients are often diagnosed at an advanced stage, and the most important thing is tumor metastasis and recurrence after treatment. In recent years, with the emergence of innovative early detection and treatment strategies and the continuous development of novel molecular therapy biomarkers 5-7, it has brought unprecedented opportunities for the clinical diagnosis and treatment of ESCC.

The mucins are a class of glycosylated proteins, which can be classified into two groups based on their structure and functions: gel-forming mucins and transmembrane mucins 8. The transmembrane mucins are widely existed in epithelial cells, with the function of providing protection and transducing signals. Abnormal expression of transmembrane mucins was found in different cancers and associated with tumor cell functional phenotype such as cell proliferation, migration and invasion 9,10. MUC15 was a member of transmembrane mucins. It was originally found from bovine milk and named PAS Ⅲ 11. MUC15 was composed of N-terminal region, transmembrane domain and a cytoplasmic region with 74 amino acids. Without typical mucin tandem repeat domain, MUC15 only contains an extracellular region with glycosylation sites. Its glycosylation density varies among different tissues and may play different roles in physiological and pathological processes. Abundant expression of MUC15 was observed in multiple tissues like thyroid, skin, placenta, lung and esophagus. MUC15 is abnormally expressed in several tumors and plays both anti-tumor and promoting effects 12-14. But till now, the role of MUC15 in ESCC remains to be investigated.

Weighted co-expression network analysis (WGCNA) is a bioinformatic method which groups highly correlated genes and analyzes the correlation between modules and clinical characteristics so as to identify candidate biomarkers or therapeutic targets. Through WGCNA, we screened for MUC15 which was down-regulated in ESCC and correlated with prognosis with TCGA and GEO datasets. Then the correlation between MUC15 and clinical characteristics or immune cells was analyzed. In 10 pairs of tumors and normal tissues, we further validated the expression of MUC15 and investigated its role in ESCC with *in vitro* and *in vivo* experiment.

## 2. Materials and methods

### 2.1 Datasets from TCGA and GEO database

The gene expression profiles of ESCC were downloaded from TCGA (https://portal.gdc.cancer.gov/). There were 81 ESCC samples and RNA-seq count data on 59427 genes. In addition, the normalized expression profiles of GSE53625 and GSE161533, gene expression profiles of ESCC from GEO were obtained. GSE53625 consisted of 179 tumor samples and 179 paired normal tissues from patients with ESCC, while GSE161533 included 28 pairs of tumor and normal tissue samples, which were studies with the GPL570 [HG-U133_Plus_2] Affymetrix Human Genome U133 Plus 2.0 Array and a list of 32059 genes were selected for the subsequent analysis. ESCC samples from TCGA and GEO were combined when prognostic analysis was performed. R package “Combat” algorithm was used to eliminate batch effects 15.

### 2.2 Weighted co-expression network construction and identification of key modules

Weighted co-expression network analysis can be used to identify candidate biomarkers or therapeutic targets. In our research, WGCNA was used to analyze differential genes in GSE53625, and the co-expression modules and key genes related to ESCC were found. We selected soft-threshold β=8 and R^2^=0.87 to build the scale-free network. Then, an adjacency matrix was created and transformed into a topological overlap matrix (TOM). A hierarchical clustering dendrogram of 1-TOM matrix was constructed to classify similar gene expression into different gene co-expression modules with a minimum number of 60 genes. Finally, we calculated the correlation between the clinical data and modules with module epigengene (ME) and the module membership (MM) to identify functional modules. The module with highest correlation coefficient and the smallest p-value was defined as the disease related 16.

### 2.3 Screening for biomarkers of ESCC

Differentially expressed genes between ESCC and normal tissues were identified through using the R package *limma* for GSE161533 and GSE53625 (|log2FC|≥2; FDR<0.05). A heatmap of top 50 differentially expressed genes plotted with R package *ggplot2.* Based on TCGA-ESCC and GSE53625, univariate Cox analysis of overall survival (OS) was performed to screen genes with prognostic value (*P*<0.05). Then, the differentially expressed genes with prognostic value were intersected with genes from interested WGCNA modules. The LASSO analysis was performed to classify the biomarkers of ESCC with *glmnet* R package,the response type was set as binomial, and the alpha was set as 1. ROC curve and AUC were used to evaluate the sensitivity and specificity of biomarkers.

### 2.4 Gene functional annotation and immune-related scores analysis

To explore possible biological functions and signaling pathways of these DEGs, Then the DEGs were inputted into the “Metascape” website for functional and pathway enrichment analysis 17. CIBERSORT was applied to estimate the relative abundances of immune cell types through gene expression profilings which further implied about the tumor immune filtration levels of ESCC 18. Then the correlation between gene expression and infiltrating immune cells was performed.

### 2.5 Analysis of clinical characteristics and Cox regression analysis

Analysis of Clinical Characteristics of ESCC and Correlation Analysis of Key Genes. The associations between the gene and clinical characteristics of ESCC, including diseases type, age, grade stage and survival data, were further analyzed. Then, the correlation matrix of these genes was plotted. Univariate and multiple Cox regression were performed to screen out prognostic factors.

### 2.6 Cell culture and collection of tissue samples

The human ESCC cell lines (TE1 and KYSE-150) were purchased from the Shanghai Cell Bank, and were cultured by using 10% FBS in DMEM (Gibco, USA) and 1% antibiotics (HyClone, USA). Ten matched pairs of fresh frozen primary tissues of ESCC and matching surrounding normal tissues were obtained from individuals with ESCC at the Changhai hospital. These patients pathologically diagnosed with ESCC and with no preoperative treatment were selected. The Changhai hospital's ethics committees approved this research (Approve number: CHEC2020-021) and all study participants signed informed consent which was conducted in accordance with the declaration of Helsinki.

### 2.7 Lentiviral vectors and transfection

MUC15 overexpression lentiviral vectors with 3×Flag tag was constructed by OBiO Technology Co. (Shanghai, China). Lentivirus infection was performed according to the manufacturer's guidelines. Stably transfected cell was screened with Puromycin (Beyotime, China).

### 2.8 Western blotting

Proteins were separated by SDS-PAGE and transferred to polyvinylidene difluoride (PVDF) membrane (Millipore, IPVH00010). The membranes were subsequently blocked with 10% non-fat milk for 1 h. The PVDF membranes were immunoblotted with anti-Flag antibody (Abcam; ab1162) diluted 1:5000 and anti-GAPDH antibody (Proteintech, HRP-60004) diluted 1:5000 at room temperature for 1 h, and then incubated with goat anti-rabbit IgG-HRP (Abcam, ab6721) with a dilution of 1:2000, and developed with a chemiluminescent reagent (EpiZyme, SQ202). Protein bands were visualized on the chemiluminescent imaging system (Bio-Rad).

### 2.9 Immunohistochemistry (IHC)

Two independent pathologists performed IHC using a modified Histo-score (H-score). The 10 ESCC tissue slides were incubated with primary MUC15 antibody (Sigma-Aldrich HPA026110). The detailed immunostaining staining procedures were performed with reference to our previous study 19.

### 2.10 Cell proliferation assay

Counting Kit-8 (CCK-8) and EdU assay were used to evaluate cell proliferation potential. In CCK-8 assay, 2.5 × 10^3^ cells were seeded into 96-well plates for 24, 48, 72 and 96 h respectively. Then, 10 µL CCK-8 solution (Beyotime, C0042) was added for 1 h. The absorbance of each sample was assessed by a microplate reader set at 450 nM. Each sample was performed for three times.

EdU assay was conducted according to the manufacturer's instructions. About 3 × 10^5^ cells were seeded in 6-well plates and maintained for 24 h. The next day, 500 µL EdU working solution (10 µM) (Beyotime, C0071S) was added to each well and incubated for 2 h. After washing with PBS for three times, cells were fixed with 4% paraformaldehyde solution (Beyotime, P0099) for 15 min, permeabilized with enhanced immunostaining permeabilization buffer (Beyotime, P0097) for 15 min, and then incubated with the click-reaction reagent for 30 min at room temperature under dark condition. At last, 1× Hoechst33342 reagent was used to counterstain the nucleus. The result was observed with a fluorescence microscope. Each sample was performed for three times.

### 2.11 Cell migration and invasion assay

Wound-healing assays were used to evaluate cell migration potential. In brief, cells were plated in 6-well plate. The scratches were observed and images were captured with a microscope at 0 and 24h pro-injury. Image-J software was employed to calculate the migration rate.

Cell invasion assays were carried out using Matrigel-coated transwell chamber system (Corning, 3496). Cells (6×10^4^) were suspended in 200 μL of serum-free medium and seeded in the upper chamber. The lower chamber contained a 500 μL medium supplemented with 10% FBS. After 48 h conventional incubation, cells on the upper filter surface were removed. Filters were then fixed in 4% paraformaldehyde (Beyotime, P0099) and stained with 0.1% crystal violet (Beyotime, C0121). All cells that invaded to the lower filter surface were counted under a microscope. Each assay was performed in triplicates.

### 2.12* In vivo* tumor growth model

BALB/c male nude mice 4-6 weeks old were purchased from Shanghai Laboratory Animal Center, Chinese Academy of Sciences (Shanghai, China). Mice were raised under pathogen-free conditions. All *in vivo* experiments were done according to approved protocols from the Institutional Animal Care and Use Committees, according to national and institutional guidelines. Briefly, to establish the xenograft model, 1×10^6^ viable cells were injected into left flank of mice. After monitoring for 4 weeks, mice were sacrificed and the tumors were harvested. Tumor weight was measured, and tumor volume was calculated by using the formula ''a×b^2^×0.5'', in which a and b represent the maximal and minimal diameters, respectively.

### 2.13 Statistical analysis

The results were presented as mean ± standard deviation (SD). Statistical evaluation of the data was performed with one-way ANOVA. Comparisons between two groups were made by using the paired t test. The P value less than 0.05 was considered statistically significant. All the statistical analyses were analyzed with SPSS version 19.0 software.

## 3. Results

### 3.1 Construction of weighted gene co-expression modules

**Figure 1:**
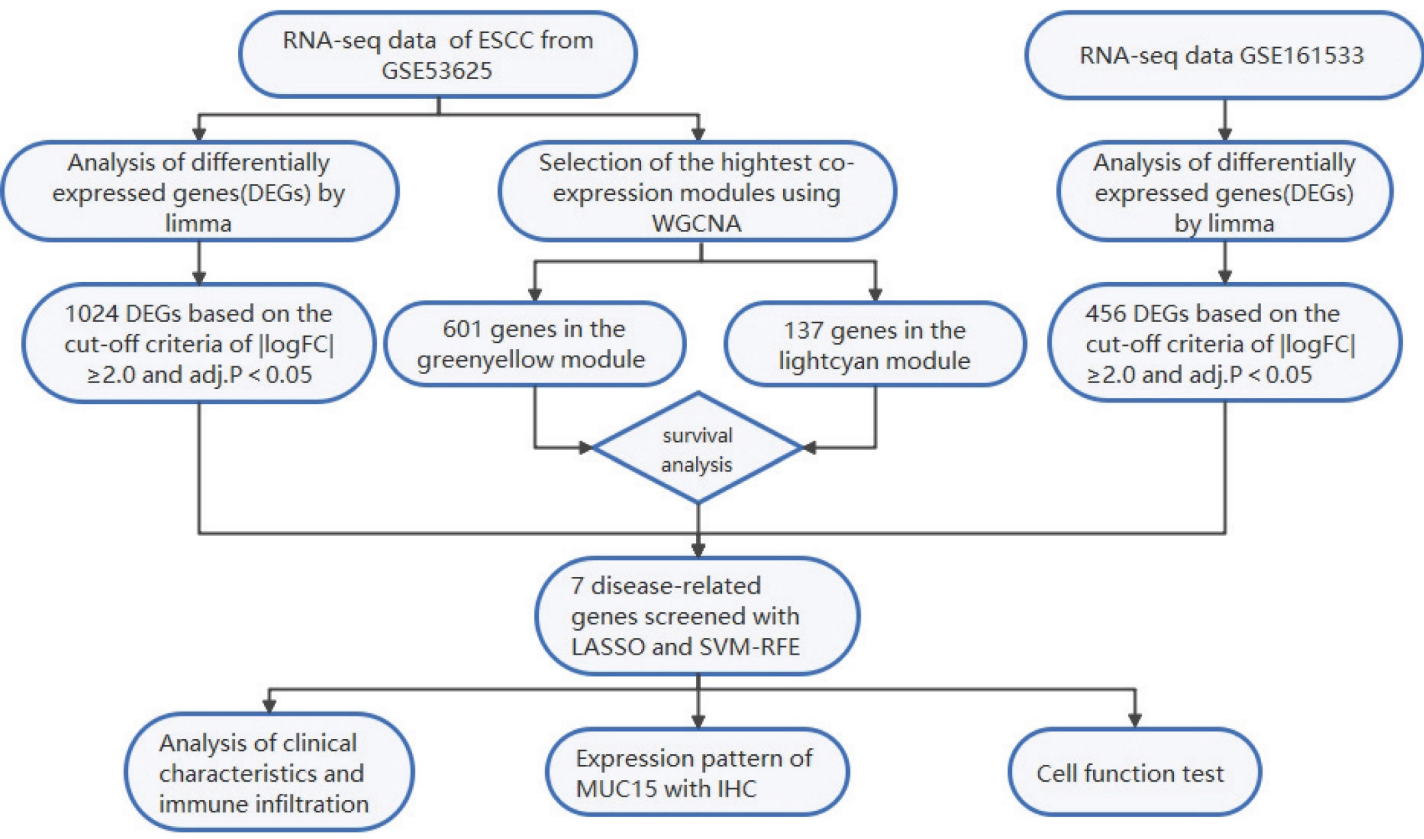
The flow chart of the study design and analysis.

**Figure 2:**
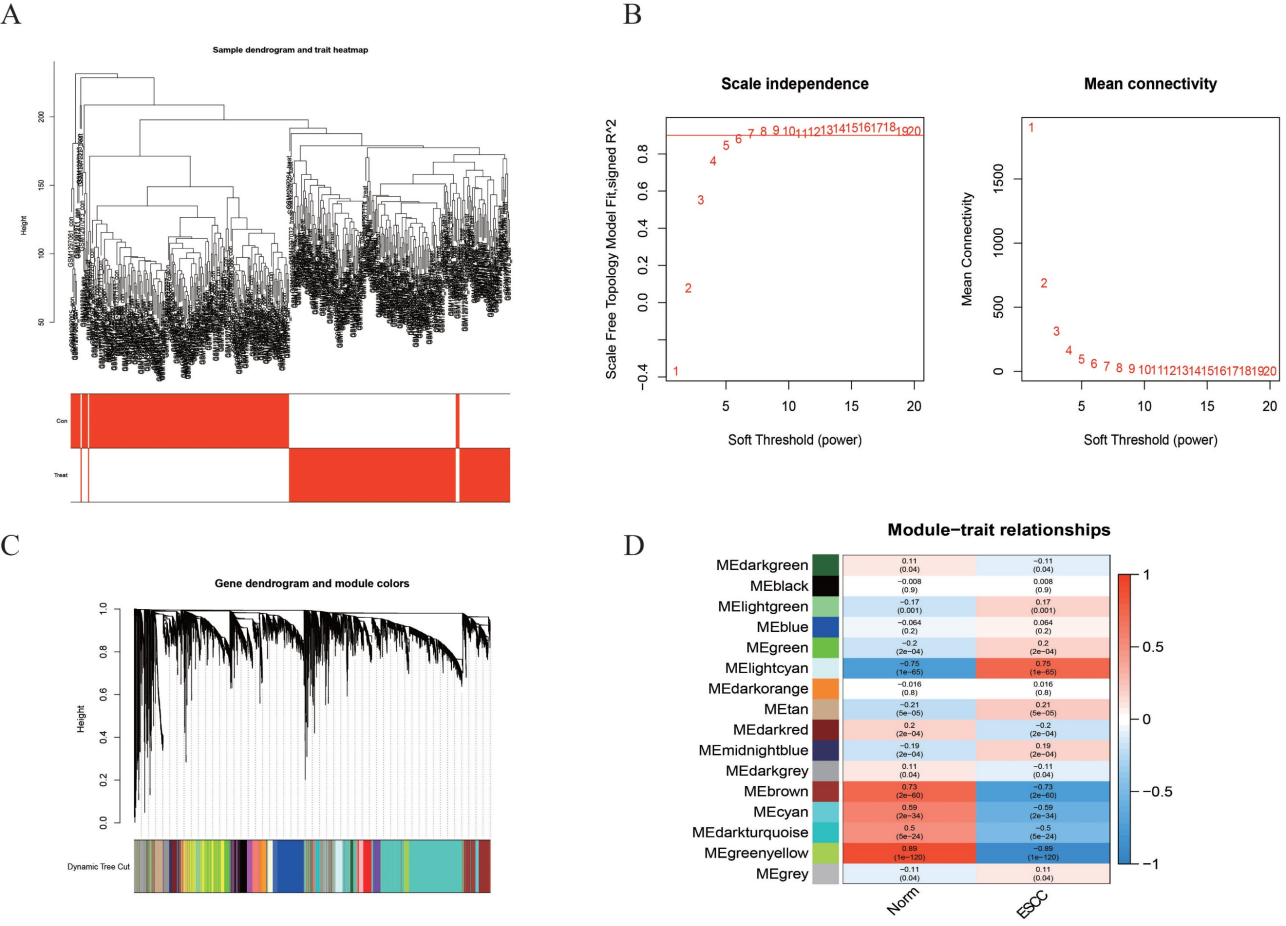
** Construction of the co-expression network.** (A) The sample dendrogram and feature heat map. (B) Soft threshold (power=8) and scale-free topology fit index (R^2^=0.87). (C) Gene hierarchy tree-clustering diagram. (D) Heatmap showing the relations between the module and ESCC.

The flowchart displays our study design is presented in Figure 1. In order to find the disease-related gene cluster, the gene co-expression network was constructed from GSE53625 dataset by WGCNA package. As a result, the sample hierarchical cluster analysis results showed good clustering among the samples (Figure 2A). The soft-threshold power was eight while the corresponding scale-free R^2^ was 0.87 (Figure 2B). The Cluster dendrogram of co-expression network modules was ordered by a hierarchical clustering of genes based on the 1-TOM matrix. Each module was assigned different colors (Figure 2C). A total of 16 modules with different colors were identified. The module-trait relationships revealed that the greenyellow and lightcyan had highest association with ESCC (greenyellow module: r=0.89, p=1e-120; lightcyan module: r=-0.75, p=1e-65) (Figure 2D).

### 3.2 Differential expression analysis and interaction with the modules of interest

**Figure 3:**
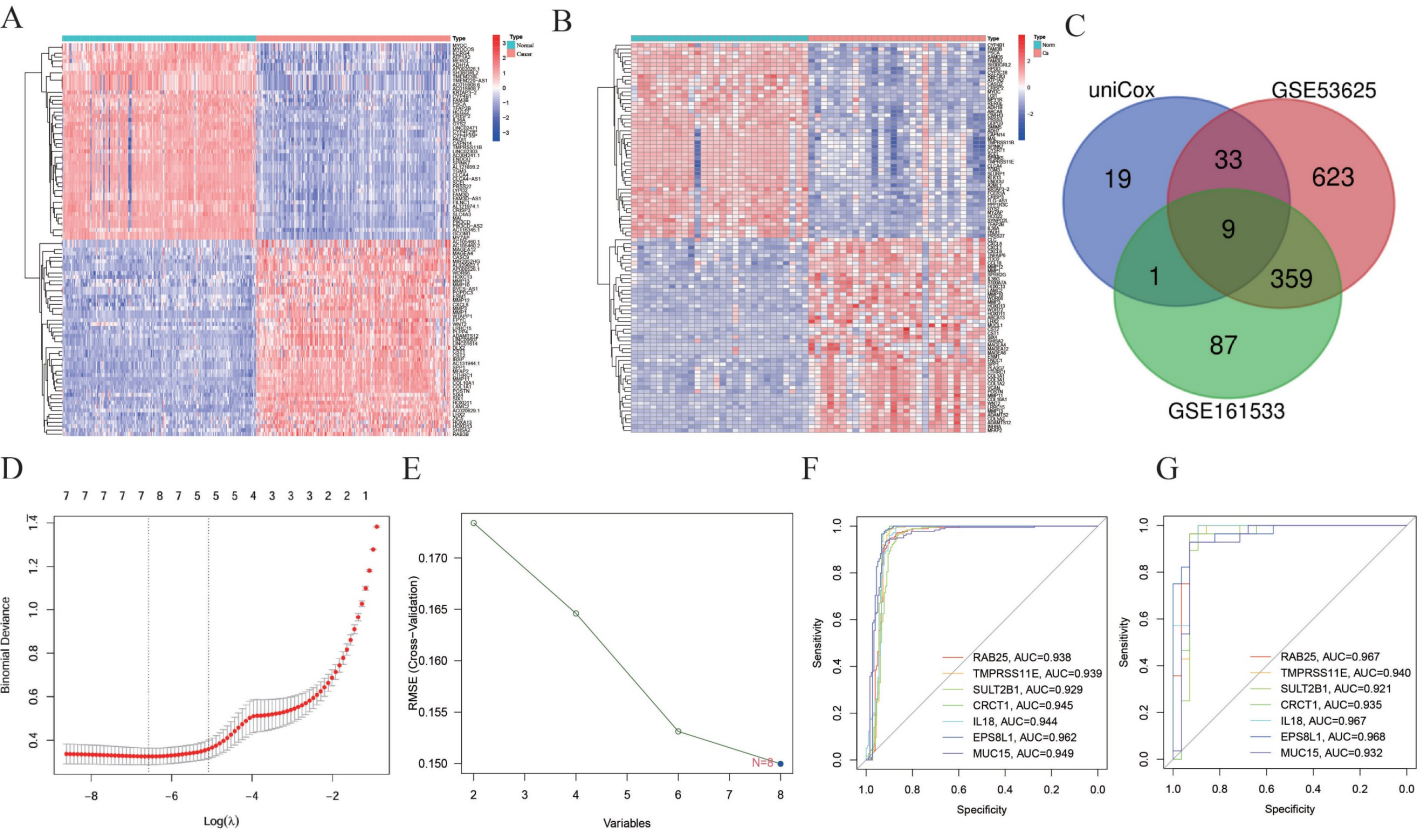
** Identification of feature genes of ESCC.** (A) Heatmap for different expressed genes (DEG) in GSE53625 and (B) DEGs in the GSE161533. (C) Venn diagram of genes extracted from DEGs and prognostic related genes. (D) LASSO regression cross-validation curve. (E) Biomarker gene screened by SVM-RFE algorithm. (F and G) AUC values of ROC analysis for the 7 feature genes in GSE53625 (F), in GSE161533 (G).

Based on the cut-off criteria of |logFC|≥2.0 and adj. *P*<0.05, a total of 1024 different expressed genes (DEG) in GSE53625 and 456 DEGs in the GSE161533 dataset were found to be dysregulated between tumors and normal tissues through *limma* package (Figure 3A and 3B). Then, functional analyses of DEGs were performed, those genes were significantly enriched in NABA_MATRISOME_ASSOCIATED, NABA_CORE_MATRISOME and Extracellular matrix organization (Supplementary Figure 1A and 1B). 601 and 137 genes were found in greenyellow and lightcyan module for survival analysis (Table S1). As a result, 62 genes were associated with prognosis (Table S2). Finally, after intersection with DEGs in GSE53625 and GSE161533, nine genes were screened (Figure 3C). Because contradiction between different expression and prognostic trend, only 8 genes were put for further analysis.

### 3.3 Selection of diagnostic markers with LASSO and SVM-RFE

Two distinct algorithms, including LASSO and SVM-RFE, were utilized to select feature genes. For LASSO regression, a total of 7 genes were selected, including CRCT1, EPS8L1, IL18, MUC15, RAB25, SERPINB2 and TMPRSS11E (Figure 3D). For the SVM-RFE algorithm, the results showed that the classifier produced the minimum error when the feature number was 8, containing all the eight genes (Figure 3E). Overall, 7 feature genes shared between the LASSO and SVM-RFE. Notably, the AUC values of ROC analysis for the 7 feature genes were all greater than 0.9 in GSE53625 (Figure 3F), which suggested that those genes might serve as diagnostic marker for ESCC patients. We further validated the reliability in GSE161533 (Figure 3G).

### 3.4 Analysis of clinical characteristics and immune infiltration

**Figure 4:**
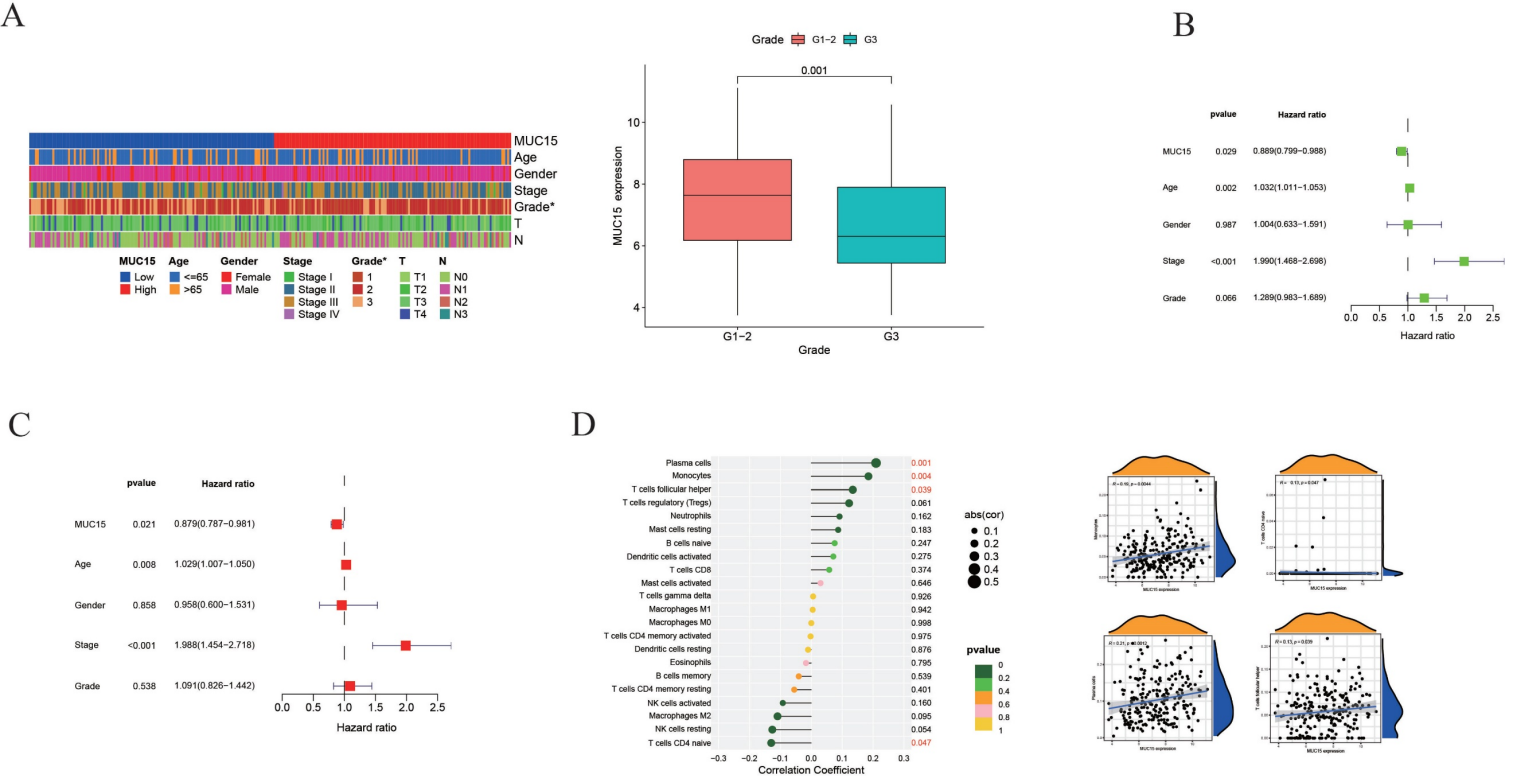
** Analysis of Clinical Characteristics and immune infiltration.** (A) Heatmap of clinicopathological features and MUC15 expression of each patient. (B and C) Univariate and multivariate Cox regression analyses of MUC15. (D) Analysis of MUC15 expression and immune infiltration.

Among 7 feature genes, MUC15 plays a role in a variety of tumors, but its role in esophageal cancer is unclear. Then, we analyzed the relationship between clinical characteristics and MUC15 expression (Figure 4A). Compared with G3 group, MUC15 expression in G1-2 group was significantly higher (*P*=0.001) while no interrelation was found between MUC15 and the other clinical characteristics (*P*>0.05). Univariate and multivariate COX regression were performed to assess whether it was independent prognostic factor for patients with ESCC. As a result, either univariate or multivariate analysis, MUC15 was still significantly correlated with overall survival (Figure 4B and 4C).

To further explore the relationship between the genes and immune infiltration, relative abundances of immune cell were calculated with CIBERSORT package. We found the MUC15 was positively correlated with plasm cells, monocytes and T cell follicular helper while it was negatively correlated with T cell CD4 naïve (Figure 4D).

### 3.5 Validation of expression pattern and cell function of MUC15

**Figure 5:**
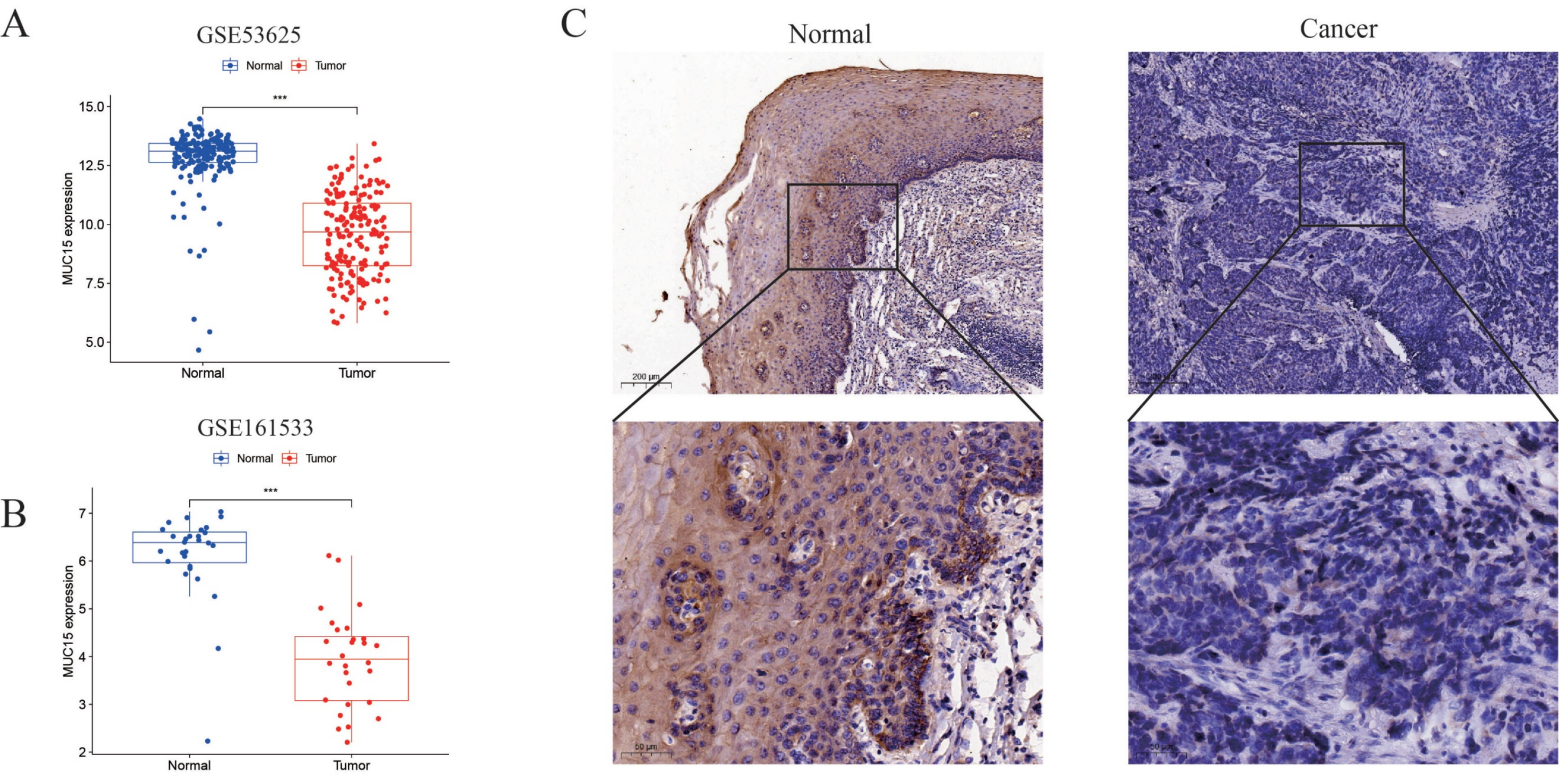
** Expression pattern of MUC15.** (A and B) Expression of MUC15 in GSE53625 and GSE161533 dataset. (C) Representative pictures of IHC staining of MUC15 in ESCC and normal tissue.

**Figure 6:**
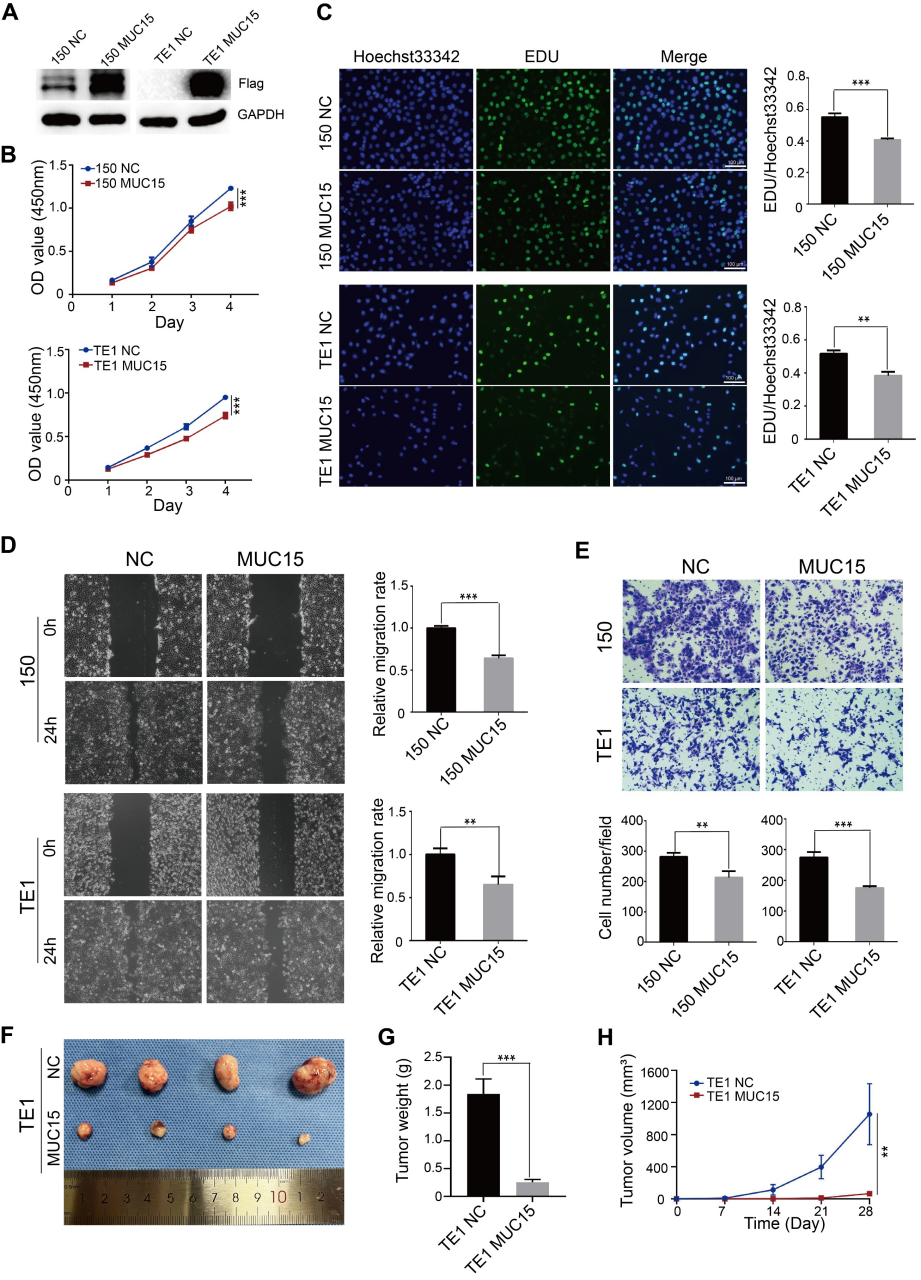
** Functional effects of MUC15 modulation on ESCC cells *in vitro* and *in vivo*.** (A) WB analysis of over-expression effect of MUC15 expression in ESCC cells (TE1 and KYSE-150); (B and C) CCK-8 (B) and EdU assay (C) evaluated the cell proliferation potential of ESCC cells (TE1 and KYSE-150) by over-expressing MUC15; (D and E) Wound-healing assay (D) and transwell assay (E) demonstrated a significant inhibiting effect in migration and invasion ability of ESCC cells (TE1 and KYSE-150) by over-expressing MUC15; (F) Xenograft model in nude mice confirmed that over-expression of MUC15 restricting tumor growth *in vivo*. (G and H) Tumor weight and volume was measured regularly. *P<0.05; **P<0.01, ***P<0.001. WB: Western blot; GAPDH: Glyceraldehyde 3-phosphate dehydrogenase; NC: Negative control; CCK-8: Counting Kit-8; OD: Optical density; EdU: 5-ethynyl-2'-deoxyuridine; ESCC: Esophageal squamous cell carcinoma.

MUC15 were down-regulated in both GSE53625 and GSE161533 dataset (Figure 5A, B). Then, the protein level of MUC15 gens was significantly lower in tumors compared with normal tissues based on IHC with 10 pair of tissues (Figure 5C). To evaluated the functional effects of MUC15 on ESCC cell, we constructed ESCC cell of stably expressing MUC15. The overexpression efficiency of MUC15 was detected by WB analysis (Figure 6A). The CCK8 and EdU assay demonstrated that overexpression of MUC15 inhibited proliferative ability of ESCC cells (Figure 6B and 6C). To explore the impact on cell motility, we performed the wound healing assay and matrigel invasion assays. Our present results showed that the mobility of cells was inhibited after overexpression of MUC15 (Figure 6D and 6E). To further validate the anti-tumor role of MUC15, we inoculated OE-MUC15 TE1 cells into nude mice. Results showed that tumors of OE- MUC15 group were significantly smaller than those from control group (Figure 6F-6H). These results confirmed that MUC15 could have the function of tumor suppressor gene and play an important role in inhibiting malignant progression of ESCC cells *in vitro* and *in vivo.*

## 4. Discussion

Esophageal squamous cell carcinoma (ESCC) is the main type of esophageal cancer (EC), which is the one of the main causes of death worldwide among all cancers 20. Despite the progression of treatment, the long-term survival of ESCC is still far from satisfaction. Thus, it is urgent to screen for potential therapeutic targets of ESCC 21. Through WGCNA and lasso regression, we found MUC15 was a biomarker of ESCC and its expression was significantly down-regulated. MUC15 was correlated with degree of tumor differentiation and could be a protective factor of survival. After calculating the relative abundances of immune cell, we found the MUC15 was positively correlated with plasma, monocytes and T cells follicular helper. Finally, we further investigated the expression of MUC15 in tissue samples and validated its inhibiting role in ESCC with cell experiments.

MUC15 is a member of transmembrane mucins and composed of N-terminal region, transmembrane domain and a cytoplasmic region with 74 amino acids 13. The extracellular domain mediates specific ligand-receptor interactions and its variability is related to normal and aberrant functions. MUC15 is expressed in multiple human tissues, especially the thyroid gland 22. Furthermore, moderate abundant expression was found in salivary gland, skin and esophagus from the data of HPA database. MUC15 is abnormal expressed in several tumors. MUC15 was significantly up-regulated in thyroid cancer 14, breast cancer 23, glioma 24, colon cancer 25 and melanoma 26 while it was down-regulated in trophoblast-like cells and hepatocellular carcinomas 27. In our study, we found MUC15 was down-regulated in ESCC through comparison of ESCC and normal tissues from TCGA and GEO dataset. Then, we further validated MUC15 protein abundance in ESCC tissue samples with IHC. We finally found MUC15 protein was down-regulated in tumor tissues and it may play anti-tumor roles.

MUC15 is abnormally expressed in a variety of tumors which suggests the dual role both pro-oncogenic and anti-tumor effect of MUC15 in tumors 13. MUC15 was with decreased expression in hepatocellular carcinoma (HCC), and its overexpression significantly suppressed EGF induced dimerization of EGFR and activation of PI3K-AKT pathway, which finally inhibited tumor cell migration and invasion 14. In thyroid cancer, tumor progression was highly correlated with the up-regualtion of MUC15. Through GPCR-cAMP and integrin-FAK, MUC15 could activate MEK-ERK pathway so as to maintain cancer cell stemness and promote metastasis 14. In our present results, MUC15 was down-regulated in ESCC tissues, and exogenous over-expression of MUC15 could significantly inhibite tumor cell proliferation, migration and invasion in TE1 and KYSE-150 cells *in vitro*. Meanwhile, the results of animal model also confirmed that over-expression of MUC15 could significantly restrict the growth of tumor cells *in vivo*. These findings suggested that MUC15 could serve as a tumor suppressor gene which inhibiting the biological functions of ESCC cells both *in vitro* and *in vivo*. Besides, our results revealed that MUC15 was positively correlated with plasm cells (P=0.001), monocytes (P=0.004) and T cell follicular helper (P=0.039) while it was negatively correlated with T cell CD4 naïve (P=0.047). Hu *et al.* reported that a prognostic risk model (including MUC15, LRFN4, ADAMTS12, MCEMP1 and HP) was significantly correlated with regulatory T cells (Tregs) in gastric cancer 28. Conte *et al.* reported that reduced mRNA expression of several mucins (MUC2, MUC12, MUC13, MUC15, MUC20, MUC21) were found in type 1 diabetes patients which companied with higher percentages of effector T cells such as T helper (Th) 1, Th17 and TNF-α^+^ T cells 29. These results suggested that MUC15 may have regulatory effects with specific types of immune cells in the tumor microenvironment. Further investigation and in-depth analysis of the correlation between MUC15 and function regulation of immune cell will help exploring novel biological targets for clinical immunotherapy of ESCC. In all, targeting MUC15 may become a promising breakthrough for individualized clinical diagnosis and treatment of ESCC in the future.

Although our present study demonstrated the potential role of MUC15 based on bioinformatics analyses and *in vitro*/*vivo* experiments, there were a number of limitations. First, the sample size and population representation which primarily relies on public databases may limit the generalizability of the findings. Future study needs to expand the sample size or validate the findings using clinical samples to enhance the generalizability of the results. Second, the long‑term effects and specific molecular mechanisms of MUC15 in ESCC need to be explored in depth. The downstream signaling pathways of MUC15 is currently unknown. Further techniques such as RNA sequencing, chromatin immunoprecipitation sequencing, immunoprecipitation and mass spectrometry should be used to investigate the direct or indirect interactions between MUC15 and key factors in downstream signaling pathways. By combining the findings of these aspects, a more comprehensive understanding of the functions of MUC15 could be expected, and these results could potentially provide a more solid experimental basis and a new strategy for clinical treatment of ESCC in the future.

## 5. Conclusions and perspectives

In conclusion, our present results identified MUC15 could serve as a tumor suppressor gene which was down-regulated in ESCC tissues and inhibited the proliferation and migration potential of ESCC cells both *in vitro* and *in vivo.* MUC15 may become a promising candidate for clinical diagnosis, individualized treatment and prognostic assessment of ESCC in the future.

## Supplementary Material

Supplementary figure and tables.
