# Supplementary figures and images for: MUC15 acts as a tumor suppressor gene which correlates with prognosis and immune infiltration in esophageal squamous cell carcinoma
Source: Int J Med Sci. 2025 Mar 21;22(8):1905–15. doi: 10.7150/ijms.108926 (PMC11983305; doi:10.7150/ijms.108926)

A

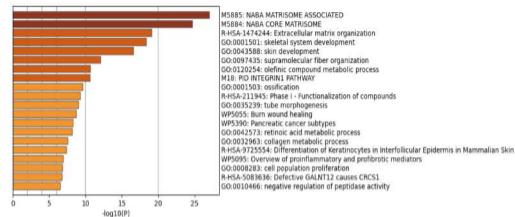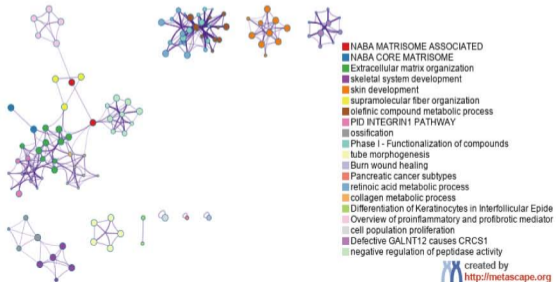

B

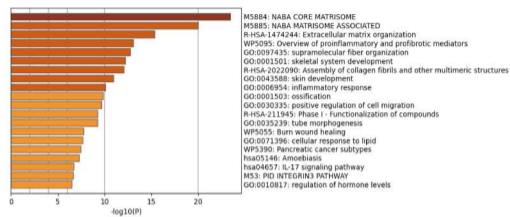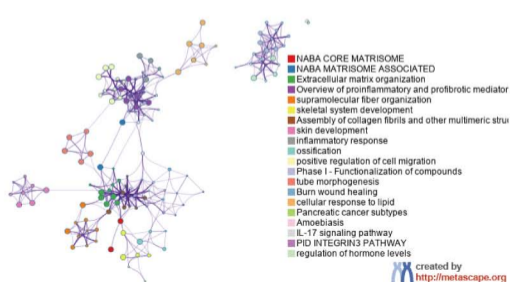

Supplement: Supplementary file 1 — Supplementary figure and tables. [file ijmsv22p1905s1.zip › Supplementary materials/Supplementary Figure 1.pdf]

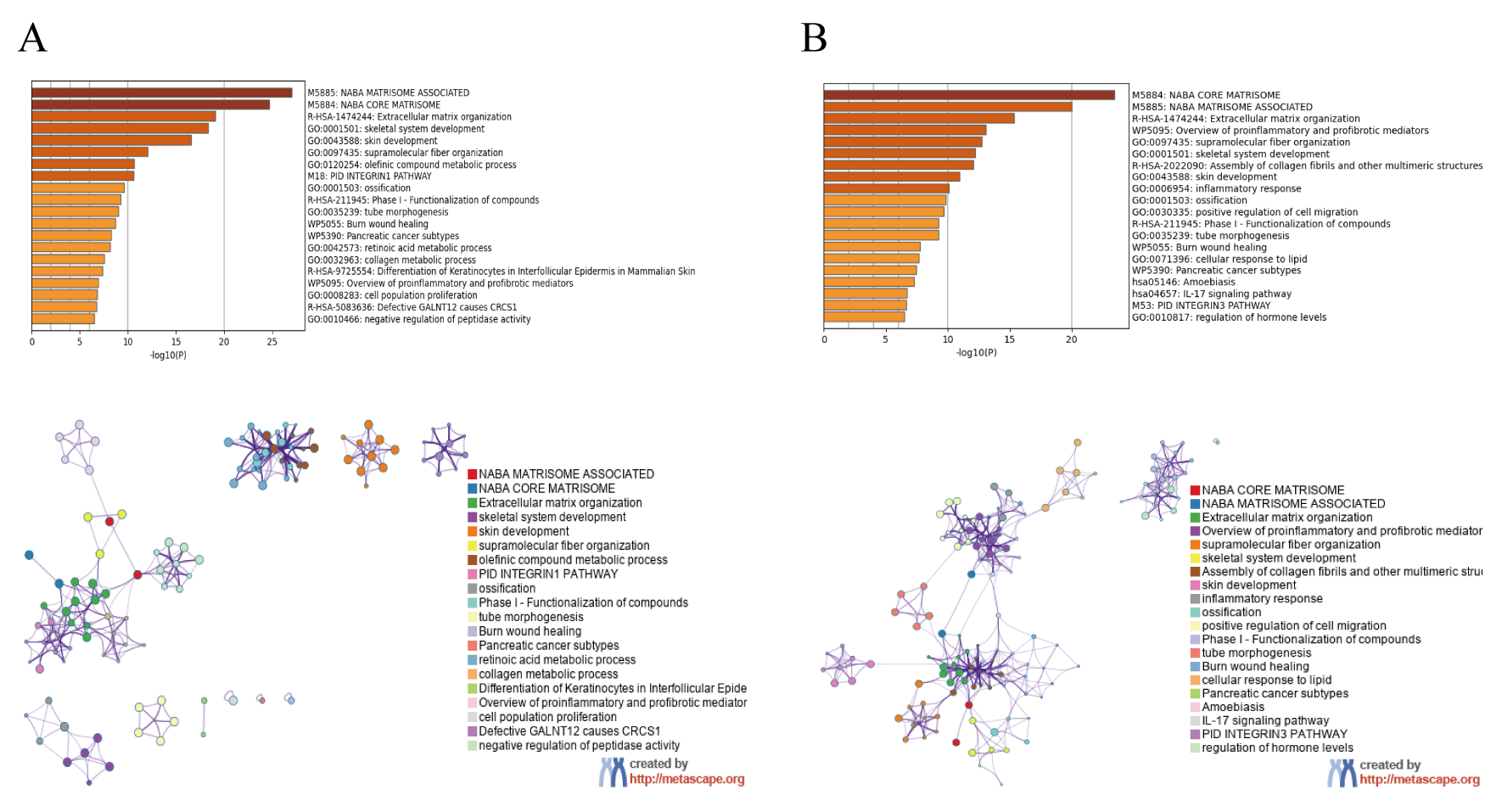

Supplement: Supplementary file 1 — Supplementary figure and tables. [file ijmsv22p1905s1.zip › Supplementary materials/Supplementary Figure 1.tif]
